# Intravascular large B-cell lymphoma presenting as rapidly progressive dementia and stroke: A case report

**DOI:** 10.1097/MD.0000000000027996

**Published:** 2021-12-03

**Authors:** Ming Wu, Yinyao Lin, Xuehong Huang, Bingjun Zhang

**Affiliations:** a Department of Neurology, Longgang District People's Hospital of Shenzhen, China; b Department of Neurology, The Third Affiliated Hospital of Sun Yat-sen University, China.

**Keywords:** case report, central nervous system, intravascular large B-cell lymphoma, rapidly progressive dementia, stroke

## Abstract

**Rationale:**

Intravascular large B-cell lymphoma (IVLBCL) is a rare form of large B-cell non-Hodgkin lymphoma. The diagnosis is challenging and frequently made at biopsy. Here we reported a case of IVLBCL limited to the central nervous system (CNS) presenting with progressive dementia and acute stroke, who was diagnosed by brain biopsy.

**Patient concerns:**

A 47-year-old woman was transferred to our hospital with a 6-month history of rapidly progressive dementia, and left limb weakness and numbness for 3 days. She was successively misdiagnosed with inflammatory demyelinating disease and stroke. Her condition deteriorated with elevated lactate dehydrogenase and multiple hyperintense lesions on the brain.

**Diagnosis:**

She was diagnosed with IVLBCL limited to the CNS by brain biopsy.

**Interventions:**

Bone marrow puncture and incisional random skin biopsy were not found neoplastic cells. Computed tomography scans were normal with no evidence of disease outside the CNS.

**Outcomes:**

The patient died due to rapid clinical aggravation.

**Lessons:**

IVLBCL limited to the CNS is an aggressive disease with high mortality. Making a timely and correct diagnosis is crucial for early appropriate treatment in IVLBCL patients.

## 1 Introduction

Intravascular large B-cell lymphoma (IVLBCL) is a rare form of large B-cell non-Hodgkin lymphoma characterized by selective proliferation of large cells within the lumen of small- and medium-sized vessels of various organs.^[1]^ IVLBCL displays a relatively high frequency of central nervous system (CNS), skin, and bone marrow involvement.^[2]^ IVLBCL limited to the CNS is infrequent, and absence of extraneural features may result in delaying diagnosis for quite some time.^[3]^ Neurologic symptoms were highly heterogeneous, including dementia, hemiparesis, seizures, myoclonus, and mental changes.^[4]^ The diagnosis is challenging and frequently made at autopsy or biopsy. Here we reported a case of IVLBCL limited to the CNS presenting with progressive dementia and acute stroke, who was diagnosed by brain biopsy.

## 2 Case presentation

**Figure 1:**
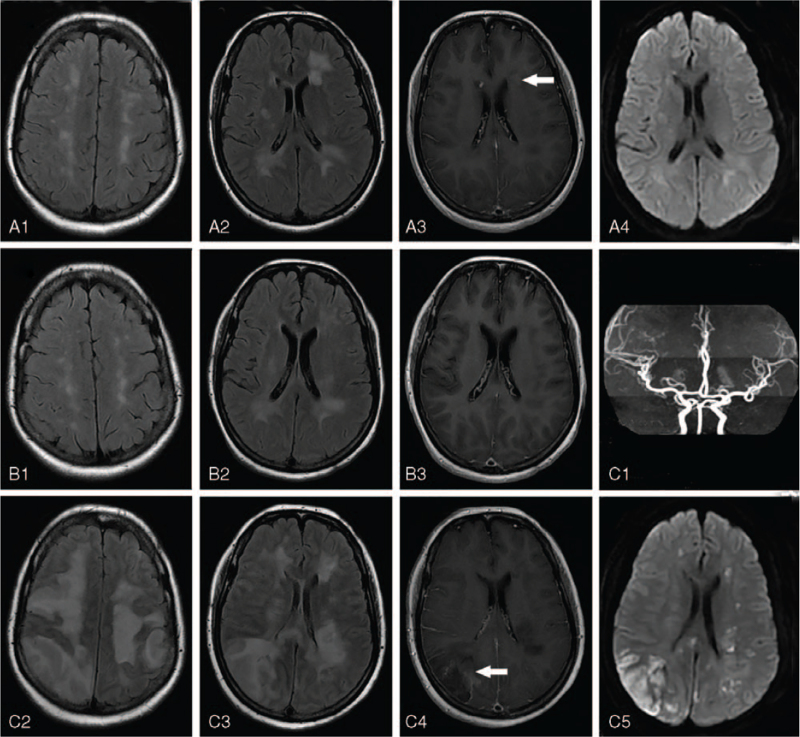
Brain magnetic resonance imaging (MRI) findings. The initial brain MRI revealed bilateral multiple hyperintense lesions in periventricular white matter, centrum semiovale, and corpus callosum on T2 fluid attenuated inversion recovery (FLAIR) images (A1, A2), while light enhancement in periventricular white matter on T1 enhanced images (A3, arrow), and dotted hyperintense lesions in diffusion weighted images (DWI) (A4). Repeated brain MRI showed the lesions decreased significantly at 2 months after discharge (B1, B2, B3). The latest brain MRI showed the lesions were enlarged and increased on T2 FLAIR (C2, C3) and DWI (C5), and the right occipital lobe lesion had open-ring enhancement on T1 enhanced images (C4, arrow). Brain magnetic resonance angiography (MRA) was normal (C1).

A 47-year-old previously healthy woman was transferred to our hospital with a 6-month history of rapidly progressive dementia (RPD), and left limb weakness and numbness for 3 days. Six months prior, she presented with progressive memory decline, calculation ability decline, and language speed slowly. She often forgot to turn on the power, and to turn off the fire when cooking. Orientation and visuoconstructive abilities were normal. There were no optic and acoustic hallucinations. No fever and limbs weakness were recorded. Mini-mental state examination (MMSE) score was 18. Vital signs, blood routine, liver and kidney function, electrolyte, lactate dehydrogenase (LDH), coagulation, tumor markers, and rheumatological examinations were normal. Brain magnetic resonance imaging (MRI) revealed bilateral multiple hyperintense lesions in periventricular white matter, centrum semiovale, corpus callosum, and cerebellum on T2 fluid attenuated inversion recovery images (Fig. 1 A1, A2), while light enhancement in periventricular white matter on T1 enhanced images (Fig. 1 A3), and dotted hyperintense lesions in diffusion weighted images (Fig. 1 A4). Spinal cord MRI did not show any abnormalities. Electroencephalograph demonstrated mild abnormality. Lumbar puncture revealed an elevated cerebrospinal fluid (CSF) leukocyte count (20 × 10^6^/L) and protein (0.89 g/L) with normal CSF glucose and chlorine. There were no malignant cells in the CSF cytology. The antibodies against nerve cell-surface antigens and intracellular antigens were negative in serum and CSF. The myelin oligodendrocyte glycoprotein, glial fibrillary acidic protein, and aquaporin-4 antibodies were not detected in serum, while the result of oligoclonal band was normal. The deoxyribonucleic acids of common virus in CSF, such as herpes simplex virus, varicella-zoster virus, cytomegalovirus, Epstein-Barr virus, were not detected. Idiopathic inflammatory demyelinating diseases were considered to be the most likely diagnosis. She was treated with intravenous methylprednisolone (1000 mg/d for 5 days, 500 mg/d for 3 days, 250 mg/d for 2 days, and 125 mg/d for 1 day), with subsequent oral methylprednisolone (20 mg/d) for 1 month and azathioprine (100 mg/d) for maintenance. Her clinical condition improved partially with MMSE score 22 at 15 days after discharge from hospital. Repeated brain MRI showed the lesions decreased significantly at 2 months after discharge (Fig. 1 B1, B2, B3).

**Figure 2:**
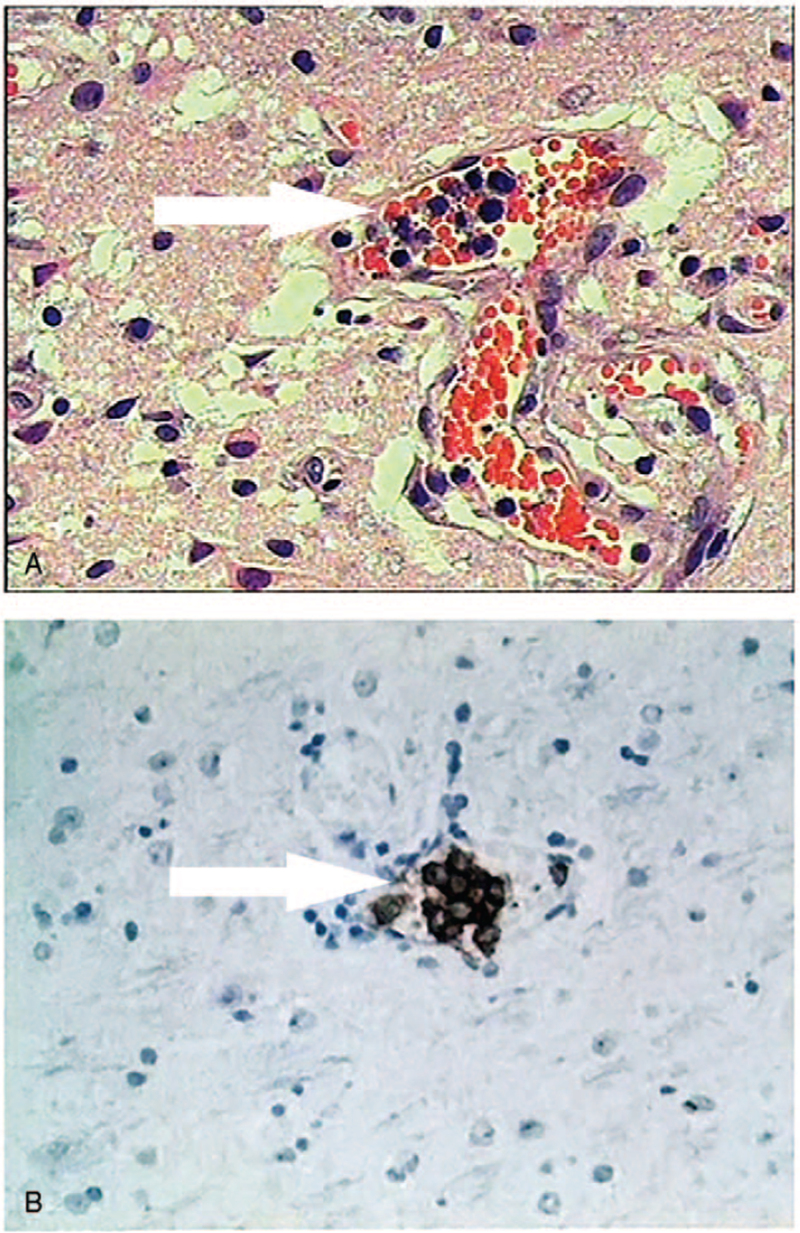
Pathological findings. Pathologic specimen showed occlusion of the small vessels by neoplastic cells with prominent nucleoli (A, arrow, H&E ×200). On immunostaining, these cells were found to be strongly positive for CD20 (B, arrow, ×200).

The patient suffered from sudden left limb weakness and numbness, and cognitive deterioration 3 days before admission. She was treated with acute stroke in local community hospital. And then, she was transferred to our hospital. On admission, she was memory decline, calculation ability decline, visuoconstructive ability decline, and disorientation with MMSE score of 9. Neurologic examination revealed left facial paralysis, dysarthria, left limbs weakness (2/5 muscle strength), and numbness with a positive Babinski sign. LDH was elevated (338 U/L). CSF revealed an elevated leukocyte count (10 × 10^6^/L) and protein (1.77 g/L) with normal glucose and chlorine. CSF samples were negative for malignant cells. After 2 days admission, she developed frequently generalized tonic-clonic seizures. Her condition deteriorated resulting in coma after 4 days admission. Brain MRI showed the lesions were enlarged and increase on T2 fluid attenuated inversion recovery and diffusion weighted images (Fig. 1 C2, C3, C5), and some lesions had open-ring enhancement on T1 enhanced images (Fig. 1 C4). Although cerebral magnetic resonance angiography (MRA) was normal (Fig. 1 C1), cerebral vasculitis was considered based on the new symptoms of stroke and seizures. Cerebral tumor was not excluded as mass-like lesions on MRI. Brain biopsy was performed on the right occipital lobe lesion. Pathologic specimen showed occlusion of the small vessels by neoplastic cells with prominent nucleoli (Fig. 2A). On immunostaining, these cells were found to be strongly positive for CD20 (Fig. 2B). Bone marrow puncture and incisional random skin biopsy were not found neoplastic cells. Computed tomography scans of the thorax, abdomen, and pelvis were normal with no evidence of disease outside the CNS. The patient was diagnosed with IVLBCL limited to the CNS, but died of brain herniation after 10 days admission.

## 3 Discussion and conclusions

The initial symptom of the present patient was RPD. RPD is a syndrome caused by numerous disease entities. Although RPD is not a rare manifestation in patients referred to neurological units, correct identification of the cause of RPD poses a diagnostic challenge.^[5]^ Former observations from prion disease reference centers reported that sporadic Creutzfeldt-Jakob disease was the most common cause for RPD followed by Alzheimer disease.^[6,7]^ Furthermore, 2 recent studies from India and Brazil reported that immune-mediated or infectious diseases of the CNS were the most common causes for RPD.^[8,9]^ Our patient did not exhibit visual hallucination, myoclonus, dystaxia, extrapyramidal signs. The patterns of brain MRI did not show strictly cortical hyperintensities (‘ribboning’) and hyperintensities in the basal ganglia. Therefore, diagnosis of Creutzfeldt-Jakob disease was not established in the patient. Because of multiple lesions in brain MRI, neurodegenerative diseases, such as Alzheimer disease, dementia with Lewy bodies, Parkinson dementia, were therefore excluded. The antibodies against nerve cell-surface antigens and intracellular antigens were negative. The results of CSF virus were normal. The evidence supporting autoimmune encephalitis or infectious diseases was insufficient. Oligoclonal band, myelin oligodendrocyte glycoprotein, glial fibrillary acidic protein, and aquaporin-4 antibodies were negative. However, the diagnosis of idiopathic inflammatory demyelinating diseases were considered, based on the dissemination of CNS lesions in space and time. The patient was treated with steroids with partial clinical and brain MRI improvement. Six months later, she developed acute left hemiparesis. Acute stroke was initially considered in the community hospital. However, her cognitive function aggravated. Repeated brain MRI showed the lesions were enlargement and increased obviously. Cerebral vasculitis and tumor were proposed. The patient was diagnosed with IVLBCL after brain biopsy.

IVLBCL is a rare and fatal type of extranodal B-cell lymphoma, characterized by the proliferation of neoplastic cells in the lumens of small vessels, especially capillaries.^[1,10]^ Two variants are described with cases in Western countries manifesting a relatively high frequency of skin rashes and multiple neurological deficits.^[11]^ Those described in Asian countries, preferentially display a typical clinical hemophagocytic syndrome, represented by bone marrow involvement, fever, hepatosplenomegaly, and thrombocytopenia.^[4]^ However, IVLBCL often presents with a set of non-specific clinical characteristics resulting from involvement of 1 or several organs. Although any organ individually or in combination can be involved, the CNS and skin are the most frequently affected organs.^[1,12]^ During the clinical course of the disease, over 60% of the patients with IVLBCL will develop neurological symptoms, including encephalopathy, stroke, seizure, RPD, myelopathy, radiculopathy, and neuropathy.^[1,13]^ However, IVLBCL limited to the CNS is an extremely rare condition as IVLBCL is frequently found with multiple organs.^[4]^ The most frequent laboratory test results of IVLBCL are anemia, high erythrocyte sedimentation rate, and elevated LDH.^[14]^ Although various patterns of abnormal features on brain MRI in patients with IVLBCL have been reported, a recent study suggested the findings on brain MRI were categorized into 4 patterns: (1) non-specific white matter lesions, (2) infarct-like lesions, (2) hyperintense lesions in the pons, and (4) meningeal thickening and/or enhancement.^[15]^ In contrast, brain MRA is not useful in the diagnosis of IVLBCL as affected cerebral vessels are too small to be directly shown on MRA.^[16]^ Given the feature of lymphoma cells in IVLBCL to remain intravascular, PET is usually negative.^[2]^ The first choice of treatment of these patients is the use of anthracycline-based on multiagent chemotherapy along with rituximab.^[4]^ Nevertheless, the prognosis of IVLBCL is poor with a high mortality rate. Our patient presented mainly with RPD and acute stroke, with elevated LDH and multiple hyperintense lesions on brain MRI. Although she was diagnosed with IVLBCL limited to the CNS by brain biopsy, she died due to rapid clinical aggravation.

We noticed several important lessons learned in this case. First, accurate diagnosis of IVLBCL limited to the CNS is challenging and still depends primarily on histopathological examination due to the lack of specific clinical manifestations, laboratory markers, and radiological features. Second, IVLBCL should be considered, when patients present with RPD and stroke with elevated LDH. Third, because appropriate treatment can improve the outcomes, making a timely and correct diagnosis is crucial for patients with IVLBCL.

## Author contributions

MW contributed to the data analysis and interpretation and drafting of the manuscript. YL and XH contributed to the data collection and analysis. BZ contributed to the manuscript's data interpretation and critical revision for important intellectual content. All authors have read and approved the manuscript.

**Conceptualization:** Ming Wu.

**Formal analysis:** Yinyao Lin.

**Investigation:** Xuehong Huang.

**Supervision:** Bingjun Zhang.

**Writing – original draft:** Ming Wu.

**Writing – review & editing:** Bingjun Zhang.
